# Self-harm: a transdiagnostic marker of psychopathology and suicide risk during the COVID-19 pandemic?

**DOI:** 10.1007/s00787-022-02044-0

**Published:** 2022-07-11

**Authors:** Michael Kaess

**Affiliations:** 1 University Hospital of Child and Adolescent Psychiatry and Psychotherapy, University of Bern, Bern, Switzerland; 2 Department of Child and Adolescent Psychiatry, Centre for Psychosocial Medicine, University Hospital Heidelberg, Heidelberg, Germany

All behaviours that are performed intentionally, and with the knowledge that they can or will result in some degree of physical or psychological injury to oneself, could be conceptualised as “self-injury” or “self-harm” [1]. Most often, self-harm is nowadays divided into two main categories: suicidal behaviour and nonsuicidal self-injury (NSSI). This division is based on the intent that is underlying the self-harming act.

In the past decades, there has been an ongoing discussion on the status of self-harm within our diagnostic classifications. For a long time, self-harm had only been considered a symptom of a few distinct disorders (e.g. depression or borderline personality disorder). In contrast, researchers had made an attempt to include both types of self-harm as independent diagnostic entities—nonsuicidal self-injury disorder and suicidal behaviour disorder—into the DSM-5 [2], which finally led to an inclusion in section 3 as diagnostic categories that warrant more research. So what exactly do we know to date about the meaning and significance of self-harm?

First, there is robust evidence that self-harm is very frequent among adolescents (i.e. a meta-analysis reported a worldwide prevalence of 16.9% with rates increasing during the past decades; [3]), and even more common among child and adolescent psychiatric patients (i.e. in more than 50% of inpatients; [4]). However, the fact that at least single episodes of self-harm occur in more than 30% of European teenagers [5] also suggests that self-harm can occur in the absence of manifest psychopathology or mental disorder, probably still indicating acute psychological strain.

Second, self-harm occurs in a variety of mental disorders and rather qualifies as a transdiagnostic marker of psychopathology. Research shows that clinical correlates of self-harm are very diverse, ranging from internalising to externalising psychopathology, and including personality pathology [6]. Severity and repetition of self-harm correlate with overall measures of increased psychopathology (e.g. [7]) and reduced quality of life suggesting that self-harm may indicate more severe mental disorder. More specifically, self-harm seems indicative of past experiences of adverse childhood experiences [8], interpersonal problems including bullying [9, 10], and emotional dysregulation [11, 12].

Third, self-harm is a marker of suicide risk. According to widely recognised theoretical concepts [13], self-harm may serve as a gateway to suicide. More specifically, individuals with repetitive self-harm may acquire the capability to attempt suicide by systematically reducing inhibition thresholds (e.g. via reduced pain sensitivity [14]). This in turn increases the likelihood for suicidal thoughts being brought into action. Indeed, empirical data show that self-harm proceeds both suicide attempts [15, 16] and completed suicide [17].

Given that self-harm is well suited as a transdiagnostic marker of psychopathology and suicide risk, the development of self-harm during the COVID-19 pandemic seems of particular interest. The COVID-19 pandemic has affected most of our and our children’s lives, and adolescents were particularly impacted by social distancing measures such as school closures and suspensions of leisure activities. In this issue, Ougrin and colleagues report on a large retrospective cohort study that investigated the development of emergency presentations due to self-harm among adolescents between 2019 and 2020, when the first wave of the COVID-19 pandemic occurred [18]. Unsurprisingly, overall presentations to emergency departments were found to have decreased significantly in March and April 2020 compared to the same time-period in 2019, during which no lockdown measures were in place. However, among all admissions, the relative proportion (but not the absolute rate) of self-harm increased. As often, these results leave some space for interpretation: On the one hand, it is possible that they point to a non-deterioration of mental health and suicide risk—at least during the first wave of the pandemic, whereby it is plausible to assume that paediatric emergencies were even more reduced compared to child and adolescent psychiatric emergencies. While this view seems to contrast the common expert opinion to date, the empirical data on the development of adolescent mental health during the first COVID-19 wave is mixed, and some studies reported overall stable mental health [19, 20], or—more differentiated—equally distributed trajectories of improvement versus deterioration of mental health during the pandemic [21]. On the other hand, several studies (e.g. [22, 23]) point towards a “real” overall deterioration of mental health during the first lockdown that could consequently explain the relative increase of self-harm emergency presentations found by Ougrin and colleagues. Interestingly, Wong and colleagues (2022) conducted further analyses on the effect of lockdown stringency in the same sample analysed by Ougrin et al., and showed that lockdown stringency—that varied considerably between sites—was positively correlated with the proportion of self-harm among emergency cases although negatively correlated with the overall rate of presentations [24]. This in turn seems to point to the possibility that lockdown measures have masked an overall deterioration of adolescent mental health across societies at the beginning of the COVID-19 pandemic. Later during the pandemic, this deterioration has consequently led to the well-known and still lasting overburdening of our mental healthcare systems.

Given that self-harm is a valid and important marker of general mental health, it will be very important to follow its development over the course of the pandemic and further on. In particular, the potential increase of adolescent self-harm as a prospective marker of suicide risk should increase our alertness for a potential suicide wave in youth that could not yet be observed during the first wave of the pandemic [25]. However, this still might occur time-delayed due to the prolonged stress of the pandemic and other critical events (e.g. the Ukraine war) worldwide.
